# Supplementary material for: Iterative improvement in the automatic modular design of robot swarms
Source: PeerJ Comput Sci. 2020 Dec 7;6:e322. doi: 10.7717/peerj-cs.322 (PMC7924708; doi:10.7717/peerj-cs.322)
Supplement: Supplemental Information 3 [file peerj-cs-06-322-s003.zip › argos3/doc/api/standalone/a00604_source.html]

ARGoS: plugins/simulator/physics\_engines/dynamics2d/dynamics2d\_differentialsteering\_control.cpp Source File


- Main Page
- Related Pages
- Namespaces
- Classes
- Files

- File List
- File Members

# plugins/simulator/physics\_engines/dynamics2d/dynamics2d\_differentialsteering\_control.cpp

Go to the documentation of this file.

```
00001 
00007 #include "dynamics2d_differentialsteering_control.h"
00008 
00009 namespace argos {
00010 
00011    /****************************************/
00012    /****************************************/
00013 
00014    CDynamics2DDifferentialSteeringControl::CDynamics2DDifferentialSteeringControl(CDynamics2DEngine& c_engine,
00015                                                                                   Real f_max_force,
00016                                                                                   Real f_max_torque,
00017                                                                                   Real f_interwheel_distance) :
00018       CDynamics2DVelocityControl(c_engine, f_max_force, f_max_torque), 
00019       m_fInterwheelDistance(f_interwheel_distance) {}
00020 
00021    /****************************************/
00022    /****************************************/
00023 
00024    void CDynamics2DDifferentialSteeringControl::SetWheelVelocity(Real f_left_wheel,
00025                                                                  Real f_right_wheel) {
00026          /*
00027           * THE DIFFERENTIAL STEERING SYSTEM
00028           *
00029           * check http://rossum.sourceforge.net/papers/DiffSteer/
00030           * for details
00031           *
00032           * Equations:
00033           *
00034           * w = (Vr - Vl) / b
00035           * v = [ ((Vr + Vl) / 2) cos(a),
00036           *       ((Vr + Vl) / 2) sin(a) ]
00037           *
00038           * where:
00039           *        a  = body orientation
00040           *        w  = body angular velocity
00041           *        v  = body center linear velocity
00042           *        Vr = right wheel velocity
00043           *        Vl = left wheel velocity
00044           *        b  = length of wheel axis
00045           */
00046       SetAngularVelocity((f_right_wheel - f_left_wheel) / m_fInterwheelDistance);
00047       Real fVelocity = (f_right_wheel + f_left_wheel) * 0.5f;
00048       CVector2 cVelocity(fVelocity * ::cos(m_ptControlledBody->a),
00049                          fVelocity * ::sin(m_ptControlledBody->a));
00050       SetLinearVelocity(cVelocity);
00051    }
00052 
00053    /****************************************/
00054    /****************************************/
00055 
00056 }
```

---

Generated on 10 Jul 2018 for ARGoS by 
 1.6.1
